# Characterization of the mitochondrial genome of *Tetrastichus howardi* (Olliff 1893) (Hymenoptera: Eulophidae)

**DOI:** 10.1080/23802359.2021.1963340

**Published:** 2021-08-19

**Authors:** Xue Tang, Baoqian Lyu, Hui Lu, Jihong Tang, Rui Meng, Bo Cai

**Affiliations:** a Provincial Key Laboratory for Agricultural Pest Management of Mountainous Regions, Institute of Entomology, Guizhou University, Guiyang, China; b Environment and Plant Protection Institute, Chinese Academy of Tropical Agriculture Sciences, Haikou, China; c Key Laboratory of Integrated Pest Management on Tropical Crops, Ministry of Agriculture and Rural Affairs, Haikou, China; d Post-Entry Quarantine Station for Tropical Plant, Haikou Customs District P. R. China, Haikou, China; e Hainan Province Engineering Research Center for Quarantine, Prevention and Control of Exotic Pests, Haikou, China

**Keywords:** Eulophidae, mitochondrial genome, *Tetrastichus howardi*, phylogenetic relationship

## Abstract

The mitochondrial genome has been widely used in the study of phylogeny and species-level evolution. Here, we sequenced and analyzed the full mitogenome of *Tetrastichus howardi*, an important natural enemy of many lepidopteran pests. The complete mitochondrial genome has 14,791 nucleotides, containing 13 protein-coding genes (PCGs), 2 ribosomal RNA genes (rRNAs), 22 transfer RNA genes (tRNAs), and a partial control region. All the 13 PCGs started with typical ATN (ATA, ATG, and ATT) codon. Among 13 PCGs, nine genes terminated with the stop codon TAA and four genes terminated with T. Our study provides information on comparative mitogenomics of Eulophidae.

*Tetrastichus howardi* (Hymenoptera: Eulophidae), an exotic pupae parasitoid of a great number of Lepidoptera pests, has a great potential for biological control (Favoreto et al. 2021; Rodrigues et al. 2021). The parasitoid was introduced into South Africa from the Philippines as a potential biocontrol agent for two important Lepidoptera pests, the *Chilo partellus* Swinhoe and *Busseola fusca* Fuller, which were serious pests of corn and sorghum in the country (Skoroszewski and Hamburg 1987; Kfir 2001). The phylogenetic relationship of the Eulophidae based on molecular and morphology analyses has been conducted, but the position of the genera in the family is not clear (Tian et al. 2021; Tang et al. 2021). In this paper, we analyzed the mitochondrial genome of the *T. howardi* for the first time, which provided valuable information for further study on molecular systematics and phylogenetic relationships within the Eulophidae.

In this study, the specimen of *T. howardi* was collected from the Environment and Plant Protection Institute, Chinese Academy of Tropical Agriculture Sciences, Hainan, China (19°59′21′′N, 110°20′9′′E) and deposited in the Post-Entry Quarantine Station for Tropical Plant, Haikou Customs District P.R. China (http://www.rdzw.net.cn/, huawei0391@163.com) under the voucher number IN07040201-0001-00010. Total genomic DNA of *T. howardi* was extracted from a single sample by CTAB method (Reineke et al. 1998). The mitogenome sequence was generated using Illumina HiSeq X TEN Sequencing System with 150 bp paired-end reads. A total of 1.17 Gb clean data was obtained and assembled by the MITObim software (Hahn et al. 2013) with the complete mt genome (MN123622) of *Tamarixia radiata* as a reference. The annotations were mainly compared with the existing mitochondrial genomes of related species, and the annotation results were confirmed by MITOS webserver (Bernt et al. 2013). The sequence was submitted to GenBank under the accession number MZ334468.

The nucleotide composition of the mitogenome sequence is 43.8% A, 41.7% T, 8.0% C, 6.5% G, with a high AT bias of 85.5%. All the genes were distributed on two coding chains, of which 26 genes were encoded on the majority strand (J-chains) and 11 genes were encoded on minority chains (N-chains). All 13 protein-coding genes get off by the conventional ATN as start codon, including six ATGs (*ATP6*, *COX1*, *COX3*, *CYTB*, *NAD4*, and *NAD6*), three ATTs (*COX2*, *NAD3*, and *NAD4L*), and four ATAs (*ATP8*, *NAD1*, *NAD2*, and *NAD5*). Nine (*ATP8*, *COX1*, *CYTB*, *NAD2*, *NAD3*, *NAD4*, *NAD4L*, *NAD5*, and *NAD6*) of 13 protein-coding genes use TAA for the stop codon, while *ATP6*, *COX2*, *COX3*, and *NAD1* genes use the incomplete stop codon, T. The length of tRNA genes ranges from 60 to 71 bp and all of them can be folded into the typical cloverleaf secondary structure, except for trnS1, which lack dihydrouridine (DHU) arm. In previous studies, there is a lack of DHU arm in the trnS1 of many insect mitochondrial genomes (Xiong et al. 2019; Tang et al. 2021). The *lrRNA* is 1320 bp in length with an A + T content of 88.1%, and the *srRNA* is 748 bp long with an A + T content of 81.9%.

Based on the concatenated amino acid sequences of 13 PCGs, the phylogenetic relationship of *T. howardi* with 22 other parasitoid and an outgroup species (*Tetranychus urticae*) was constructed by the neighbor-joining method using MEGA 7.0 software (Sudhir et al. 2016). The result showed that *T. howardi* was closely related to *T. radiata* (Figure 1), which agrees with the conventional classification.

**Figure 1.**
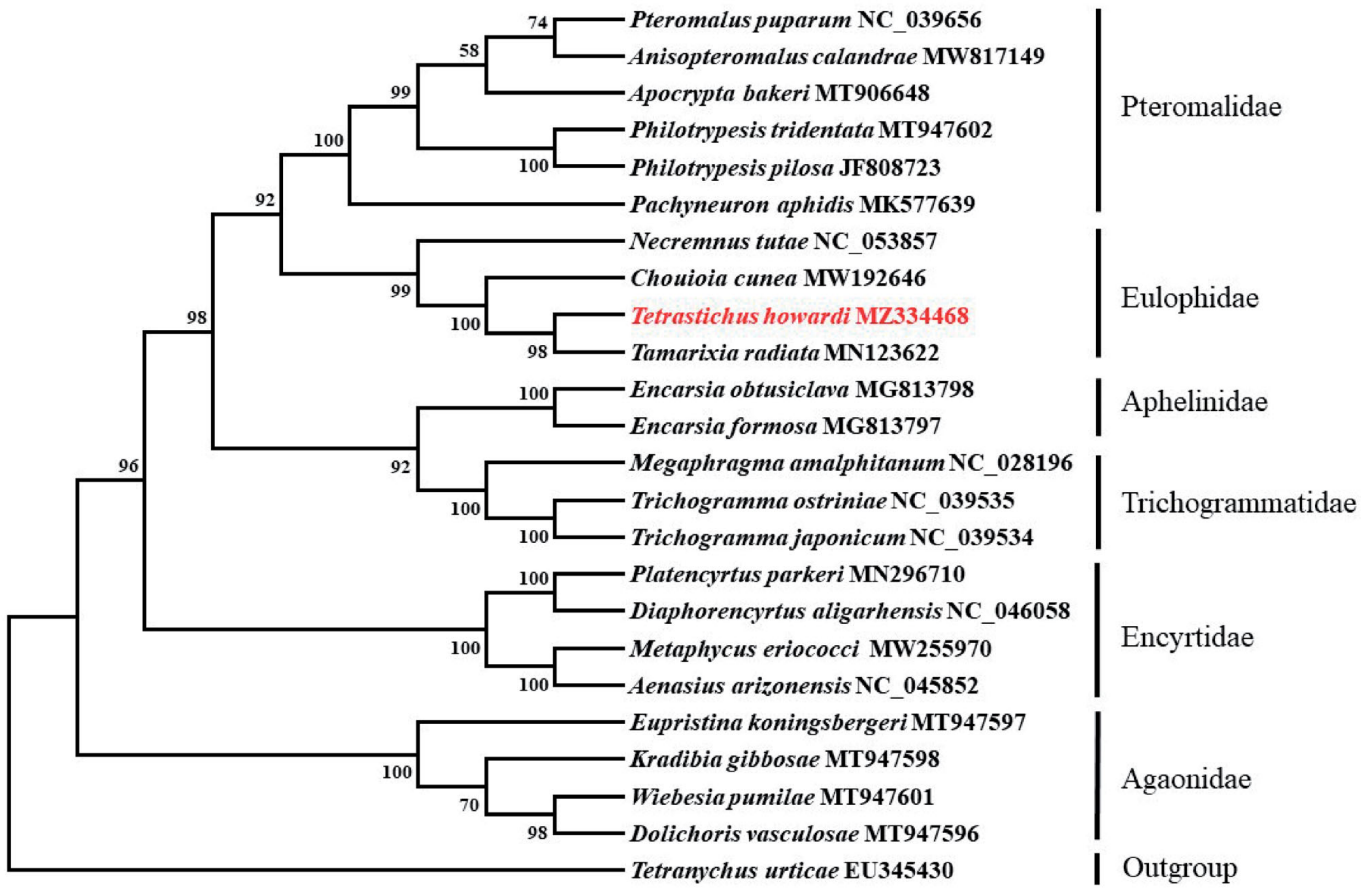
Phylogenetic tree showing the relationship between *Tetrastichus howardi* and 22 other parasitoids based on neighbor-joining method. GenBank accession numbers of each species were listed in the tree. The parasitoid determined in this study has been marked red. Bootstrap values are shown above the nodes.

## Data Availability

The genome sequence data that support the findings of this study are openly available in GenBank of NCBI at (https://www.ncbi.nlm.nih.gov/) under the accession no. MZ334468. The associated BioProject, SRA, and Bio-Sample numbers are PRJNA737393, SRR14812766, and SAMN19693530, respectively.
